# Comparative Study of Esophageal Stent and Feeding Gastrostomy/Jejunostomy for Tracheoesophageal Fistula Caused by Esophageal Squamous Cell Carcinoma

**DOI:** 10.1371/journal.pone.0042766

**Published:** 2012-08-13

**Authors:** Yen-Hao Chen, Shau-Hsuan Li, Yi-Chun Chiu, Hung-I Lu, Cheng-Hua Huang, Kun-Ming Rau, Chien-Ting Liu

**Affiliations:** 1 Department of Hematology-Oncology, Kaohsiung Chang Gung Memorial Hospital and Chang Gung University College of Medicine, Kaohsiung, Taiwan; 2 Department of Hepato-Gastroenterology, Kaohsiung Chang Gung Memorial Hospital and Chang Gung University College of Medicine, Kaohsiung, Taiwan; 3 Department of Thoracic & Cardiovascular Surgery, Kaohsiung Chang Gung Memorial Hospital and Chang Gung University College of Medicine, Kaohsiung, Taiwan; Johns Hopkins University, United States of America

## Abstract

**Background:**

A malignant tracheoesophageal/bronchoesophageal fistula (TEF) is a life-threatening complication of esophageal squamous cell carcinoma. A feeding gastrostomy/jejunostomy had been the most common treatment method for patients with TEF before the era of stenting. The aim of this retrospective study is to compare the prognosis of esophageal squamous cell carcinoma patients with TEF treated with an esophageal metallic stent to those treated with a feeding gastrostomy/jejunostomy.

**Methods:**

We retrospectively reviewed a total of 1011 patients with esophageal squamous cell carcinoma between 1996 and 2011 at Kaohsiung Chang Gung Memorial Hospital, and 86 patients with TEF (8.5%) were identified. The overall survival and other clinical data were compared between 30 patients treated with an esophageal metallic stent and 35 patients treated with a feeding gastrostomy/jejunostomy.

**Results:**

Among the 65 patients receiving either an esophageal metallic stent or a feeding gastrostomy/jejunostomy, univariate analysis showed that treatment modality with an esophageal metallic stent (P = 0.007) and radiotherapy treatment after fistula diagnosis (P = 0.04) were predictive of superior overall survival. In the multivariate comparison, treatment modality with an esophageal metallic stent (P = 0.026, odds ratio: 1.859) represented the independent predictive factor of superior overall survival. There were no significant differences between groups in mean decrease in serum albumin or mean body weight loss. Compared to the feeding gastrostomy/jejunostomy group, a significantly higher proportion of patients in the stenting group (53% versus 14%, P = 0.001) were able to receive chemotherapy within 30 days after fistula diagnosis, indicating better infection control in the stenting group.

**Conclusions:**

Compared with a feeding gastrostomy/jejunostomy, an esophageal metallic stent significantly improves overall survival in patients with malignant TEF in our retrospective analysis. Esophageal metallic stent placement may be considered the first-line of treatment for patients with malignant TEF.

## Introduction

**Figure 1:**
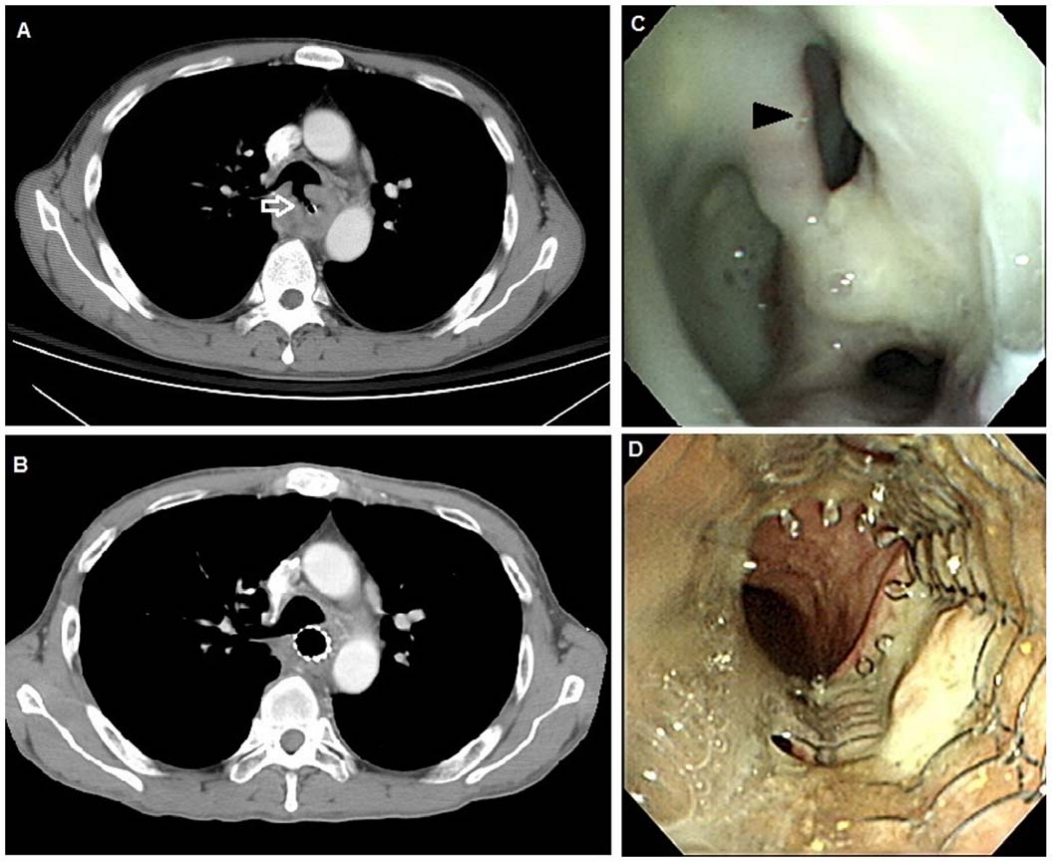
Representative images before and after esophageal metallic stent placement. A. Computed tomography of the chest obtained before stent placement showed a tracheoesophageal fistula (arrow). B. Computed tomography of the chest obtained after stent placement showed a metallic stent in the esophagus covering the tracheoesophageal fistula. C. Before stent placement, endoscopic picture showed a protruding mass with a hole in the esophagus, suggesting esophageal cancer with a tracheoesophageal fistula (arrowhead). D. Endoscopic picture of an esophageal metallic stent in place one month after insertion.

A malignant tracheoesophageal/bronchoesophageal fistula (TEF) is a pathological communication between the esophagus and the respiratory tract; it is a serious and life-threatening complication of esophageal cancer (Figure 1). 5%–10% of patients with esophageal cancer contract TEF [1], [2]. Most TEFs are caused by a tumor invasion or as a complication of cancer therapies, such as radiotherapy or chemotherapy [2], [3]. Malnutrition, frequent aspiration to the airway and repeated pneumonia episodes can lead to rapid deterioration, and the patient will soon die of respiratory failure if not treated in time. Most patients died from respiratory infections and poor nutrition within 3–4 months [2], [4].

Treatment of the malignant TEF is usually palliative and involves restoration of the swallowing mechanism and prevention of aspiration, including surgical resection/repair of the fistula, esophageal bypass, feeding gastrostomy/jejunostomy, stenting, best supportive care, or radiotherapy [1], [2], [5], [6]. Surgical intervention, such as surgical resection/repair of the fistula, is seldom performed nowadays because it carries high mortality and morbidity [6] and thus is only executed in a small number of experienced centers. A feeding gastrostomy/jejunostomy had been considered the ultimate choice to treat TEF before the era of stenting because gastrostomy/jejunostomy can at least partially palliate the respiratory symptom and establish a route of nutritional supply [5], [7], [8], [9], [10]. Hu et al. [5] reported that 9 of 35 patients (26%) with malignant tracheoesophageal/bronchoesophageal fistula received gastrostomy. In the series of Choi et al. [9], gastrostomy was performed in 20 of the 52 esophageal squamous cell carcinoma patients (38%) with esophagorespiratory fistula. Since the 1990s, esophageal intubation with stent prostheses has gradually developed. Several types of covered expandable metallic stents have been used with higher rates of complete closure of TEF, which can avoid certain complications, such as hemorrhage, perforation, pressure necrosis, food impaction, stent dislocation, occlusion, and migration [3], [11], [12], [13], [14], [15]. In Taiwan, feeding gastrostomy/jejunostomy and stenting have become the most common treatment modalities for TEF. Previous studies reported that stent prostheses implantation could improve fistula closure, symptoms of respiratory tract, and quality of life [1], [5], [11], [16], [17], [18]. However, to the best of our knowledge, compared with the feeding gastrostomy/jejunostomy, the survival benefit of esophageal metallic stent has not yet been well documented. Thus, the aim of this study is to compare the prognosis of patients with malignant TEF who receive an esophageal metallic stent to those who receive a feeding gastrostomy/jejunostomy.

**Figure 2:**
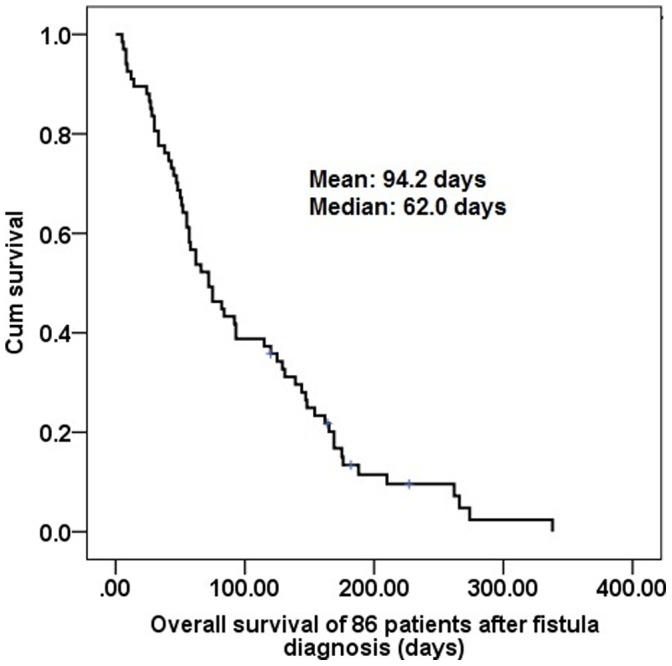
Overall survival of all 86 ESCC patients after fistula diagnosis. ESCC: esophageal squamous cell carcinoma.

**Table 1:** Treatment modality in 86 esophageal squamous cell carcinoma patients with malignant tracheoesophageal/bronchoesophageal fistula.

Treatment	No. of patients (%)
Esophageal metallic stent	30 (35%)
Feeding gastrostomy/jejunostomy	35 (41%)
Best supportive care	19 (22%)
Surgery*	2 (2%)
Total	86 (100%)

* fistula resection and repair in one patient and esophageal diversion in the other.

## Materials and Methods

### Patient Selection

1011 patients with esophageal squamous cell carcinoma at Kaohsiung Chang Gung Memorial Hospital between January 1996 and December 2011were retrospectively reviewed. Diagnoses of TEFs were established using upper gastrointestinal endoscopy, or bronchoscopy, or upper gastrointestinal barium series. Patients suspected as having TEF on a computed tomography (CT) of the chest were excluded if the fistula was not proven by one of the above mentioned methods. The tumor stages were determined according to the 7^th^ American Joint Committee on Cancer (AJCC) staging system. Of these 1011 patients, 86 (8.51%) esophageal squamous cell carcinoma patients with TEF were identified.

**Table 2:** Clinical features of 65 esophageal squamous cell carcinoma patients with malignant tracheoesophageal/bronchoesophageal fistula receiving an esophageal metallic stent or a feeding gastrostomy/jejunostomy.

Parameters	No. of patients		
	Esophageal metallicstent (n = 30)	Feeding gastrostomy/jejunostomy (n = 35)	P value
Age (Mean ± SD): 50.29±9.52	51.20±11.08	49.51±8.04	0.49
7^th^ AJCC stage			
IIIc	19	23	0.84
IV	11	12	
Respiratory location of the fistula			
Above carina	15	20	0.57
Below carina	15	15	
Primary location of the tumor			
Upper	5	8	0.53
Middle/Lower	25	27	
Radiotherapy after fistula			
Absent	16	25	0.13
Present	14	10	
Radiotherapy before fistula			
Absent	14	16	0.94
Present	16	19	

SD, standard deviation;

* Statistically significant. X^2^ test, Fisher’s exact test or t test was used for statistical analysis.

In our institution, esophageal metallic stents became available after 2000 and were the first priority when TEF was diagnosed. However, an esophageal metallic stent was not reimbursed by our health-insurance system in Taiwan. If patients refused a stent due to economic problems or their own choice, a feeding gastrostomy/jejunostomy, which was covered by our health-insurance system, was suggested. If patients were medically unfit to receive a feeding gastrostomy/jejunostomy or refused a feeding gastrostomy/jejunostomy, the best supportive care, including fasting, antibiotics, and parenteral nutrition support, was provided.

**Table 3:** Results of univariate log-rank analysis of prognostic factors for overall survival in the 65 esophageal squamous cell carcinoma patients with malignant tracheoesophageal/bronchoesophageal fistula who received an esophageal metallic stent or feeding gastrostomy/jejunostomy.

		Overall survival (days)		
Factors	No. ofpatients	Mean	Median	P value
Treatment modality				
Esophageal metallic stent	30	144.8	125.0	0.007*
Feeding gastrostomy/jejunostomy	35	76.6	55.0	
7^th^ AJCC stage				
IIIc	42	108.0	82.0	0.65
IV	23	96.7	66.0	
Respiratory location of the fistula				
Above carina	35	81.1	55.0	0.062
Below carina	30	124.9	93.0	
Primary location of the tumor				
Upper	13	105.6	93.0	0.85
Middle/Lower	52	102.7	66.0	
Age				
<50 y/o	32	115.6	84.0	0.33
≥50 y/o	33	93.3	58.0	
Radiotherapy after fistula				
Absent	41	87	62.0	0.04*
Present	24	142.6	84.0	
Radiotherapy before fistula				
Absent	30	124.5	72.0	0.06
Present	35	87	75.0	

* Statistically significant.

**Figure 3:**
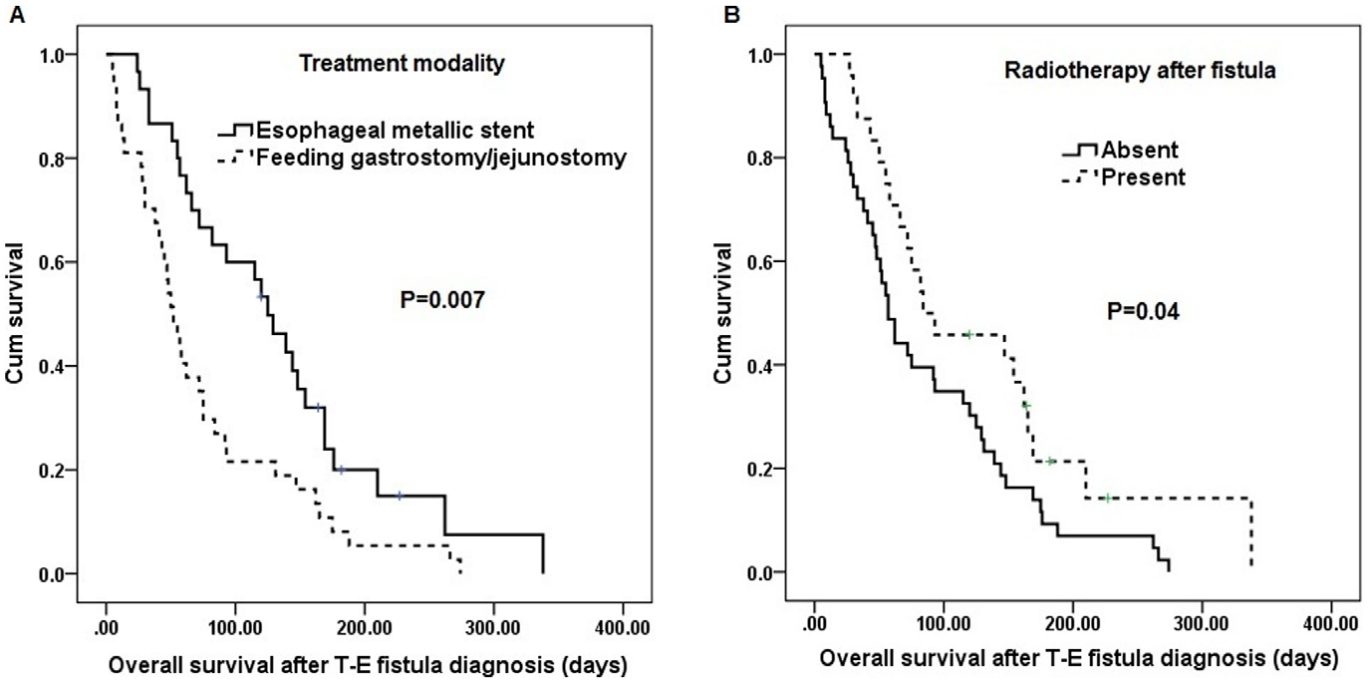
Survival curves of 65 ESCC patients according to the treatment modality(A) and radiotherapy after fistula(B). ESCC: esophageal squamous cell carcinoma.

### Esophageal Metallic Stent Placement

Before stent placement, the site of the tracheoesophageal fistula (TEF) was evaluated via upper gastrointestinal endoscopy. All patients received a topical anesthetic lidocaine hydrochloride, which was administered to the pharynx via aerosol spray. A guide wire (Hydra Jagwire; Boston Scientific Corporation, Natick, MA, USA) was inserted through the endoscope (GIF-Q240; Olympus Optical Corporation, Tokyo, Japan), passing the tumor into the distal portion of the esophagus or stomach. A covered metallic stent (Ultraflex; Boston Scientific Corporation, Natick, MA, USA) was placed via a guidewire under fluoroscopy. The size of the stent, 10 cm, 12 cm or 15 cm in length, was chosen according to tumor length and fistula location. After the length of the tumor was estimated, a stent was placed to dilate the tumor narrowing and cover the fistula opening (Figure 1).

**Figure 4:**
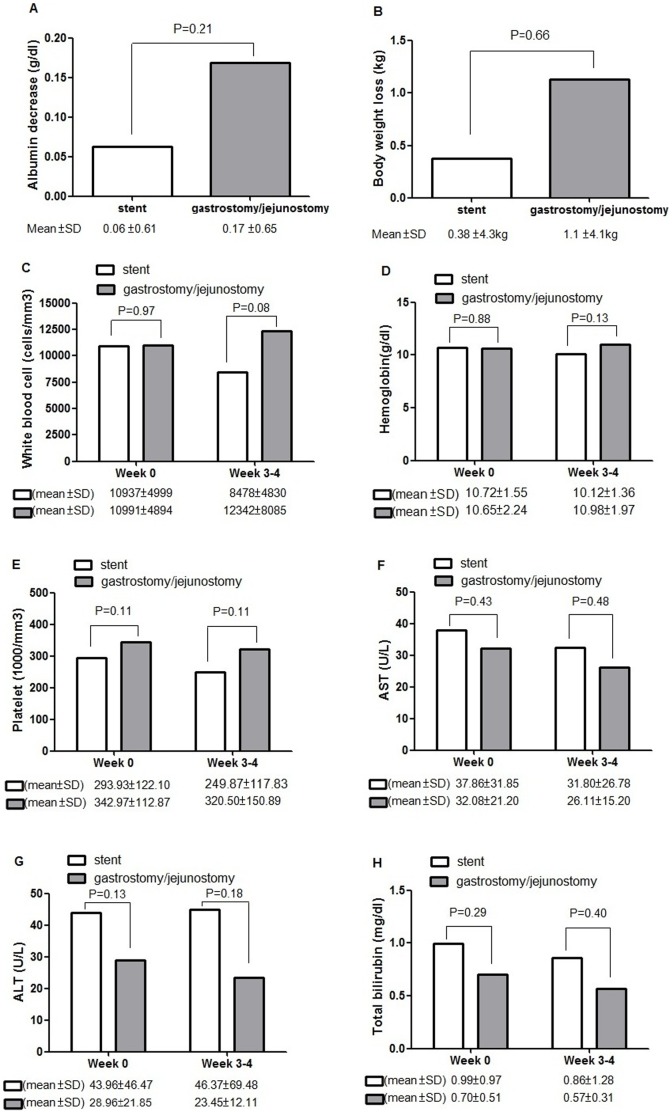
Nutrition status and other laboratory data in stenting and feeding gastrostomy/jejunostomy groups. A. Serum albumin decrease was determined by the formula: [serum albumin level within 1 week before fistula diagnosis – serum albumin level at 3–4 weeks after fistula diagnosis]. There was no significant difference (P = 0.21) between the two groups in mean decrease in serum albumin. B. Body weight loss was determined by the formula: [body weight within 1 week before fistula diagnosis – body weight at 3–4 weeks after fistula diagnosis]. There was no significant difference (P = 0.66) between the two groups in mean body weight loss. C–H. There were no significant differences between stenting and feeding gastrostomy/jejunostomy groups in white blood cell(C), hemoglobin(D), platelet(E), AST(F), ALT(G), and total bilirubin(H) within the week before fistula diagnosis (Week 0) or at 3–4 weeks after fistula diagnosis (Week 3–4).

### Tracheal Stent Placement

Before stent placement, the tracheoesophageal fistula (TEF) site and the extent of the tracheal obstruction are both evaluated via a flexible bronchoscopy and computed tomography of the chest. If the fistula tract is located 2 cm above the carina, a self-expandable Ultraflex tracheobronchial stent (Boston, Scientific) or Montgomery T-tube implantation is favored. If the carina is involved, a Novatech Dumon™ Silicone Y-stent implantation is the most common treatment choice. Tracheal stents are implanted using a rigid bronchoscopy (EFER-Dumon, Germany) under general anesthesia in all patients. The estimated tracheal diameter for stent size selection is extrapolated from the external diameter of the largest rigid bronchoscope that is able to pass through the involved trachea. The length of the stent is based on the extent of tracheal narrowing and the size of the fistula. All placed stents were at least 1 cm longer than the fistula in order to cover the opening of the fistula.

**Table 4:** Clinical retrospective series of malignant tracheoesophageal/bronchoesophageal fistulae.

Investigators	Year	Age	Primary tumor	No. of pts	Survival	Comments
Martini et al [2]	1970	58.6(mean)	ESCC (93) EAC(2) Others (16)	111	1-mth survival rate:60%3-mth survival rate:21%	Gastrostomy 39(35%), supportive care 39 (35%),bypass 10(9%), exclusion 12(11%),esophagectomy 2(2%).
Gudovsky [4]	1993	N/A	Esophagus(5)Others (6)	11	N/A	Operative mortality in 3 esophageal cancer pts
Morgan et al [3]	1997	60.3(mean)	ESCC (18)EAC (2)	20	65.3 days(mean)	Stents in all 20 pts
Hu et al [5]	2009	57.7(mean)	ESCC (35)	35(All) 17(stent)9(gastrostomy)	N/A 93 days(median)62 days(median)	Stent improves health-related quality of life
Choi [9]	2010	61(median)	ESCC (52)	52	56 days(median)	Stent 21(40%), gastrotomy 20(38%),esophagectomy 4(8%), bypass 1(2%)
Our series	2012	51.5(mean)	ESCC (86)	86(All) 30(stent)35(gastrostomy/jejunostomy)	62 days(median)125 days(median)55 days(median)	Stent improves overall survival, compared togastrostomy/jejunostomy

ESCC, esophageal squamous cell carcinoma; EAC, esophageal adenocarcinoma; N/A, not available (data not available in the publication); pts, patients.

### Nutrition Status and Other Laboratory Data Evaluation

Serum albumin level and body weight were used as surrogate markers for patients’ nutrition status. Serum albumin level and body weight within the week before fistula diagnosis and 3–4 weeks after fistula diagnosis were retrospectively recorded. Serum albumin decrease was determined by the formula: [serum albumin level within the week before fistula diagnosis – serum albumin level 3–4 weeks after fistula diagnosis]. Body weight loss was determined by the formula: [body weight within the week before fistula diagnosis – body weight 3–4 weeks after fistula diagnosis]. Besides, other laboratory data including the complete blood count (CBC), and liver function within the week before fistula diagnosis and 3–4 weeks after fistula diagnosis were also retrospectively recorded.

### Statistical Analysis

Statistical analyses were performed using the SPSS 17 software package. The chi-square test, Fisher’s exact test, and *t*-test were used to compare data between the two groups. Overall survival was calculated from the date of fistula diagnosis until death or the last follow-up. The Kaplan–Meier method was used for univariate survival analysis, and the difference between survival curves was tested by a log-rank test. In a stepwise forward fashion, parameters with P values <0.05 at univariate level were entered into Cox regression model to analyze their relative prognostic importance. For all analyses, two-sided tests of significance were used with P<0.05 considered significant.

### Ethics Statement

The retrospective analysis was approved by Chang Gung Medical Foundation Institutional Review Board. Written informed consent of the patients or their family was not judged necessary for this kind of retrospective study by Chang Gung Medical Foundation Institutional Review Board.

## Results

### Patient Characteristics

Of the 1011 patients with esophageal squamous cell carcinoma, 86 (8.51%) esophageal squamous cell carcinoma patients with TEF were identified. There were 83 men and 3 women with a mean age of 51.48 years (range: 35 to 80 years). When fistula was diagnosed, 58 patients were AJCC 7^th^ stage IIIC, and the other 28 patient were AJCC 7^th^ stage IV. The median survival from the diagnosis of the fistula was 62 days (Figure 2).

According to their treatment modalities, the 86 patients were divided into four groups according to their treatment modalities: esophageal metallic stent (n = 30), feeding gastrostomy/jejunostomy (n = 35), best supportive care (n = 19), and surgery (n = 2) (Table 1). In the surgery group, one patient received esophageal resection and repair for fistula, and the other underwent esophageal diversion. Compared with the stenting group or feeding gastrostomy/jejunostomy group, the best supportive care group has significantly inferior overall survival (P = 0.019, data not shown).

### Survival Comparison between Patients Treated with an Esophageal Metallic Stent and those with a Feeding Gastrostomy/Jejunostomy

There were 65 patients who received an esophageal metallic stent or a feeding gastrostomy/jejunostomy. The overall survival rates and other clinical data were compared between the stenting group (n = 30) and the feeding gastrostomy/jejunostomy group (n = 35). All 65 patients were men with a mean age of 50.29 years (range: 35 to 80 years). When fistula was diagnosed, 42 patients were AJCC 7^th^ stage IIIC, and 23 patients were AJCC 7^th^ stage IV. There was no significant difference in clinical features between these two groups, including age, AJCC 7th stage, location of the fistula, primary location of the tumor, radiotherapy history before fistula diagnosis, and radiotherapy treatment after fistula diagnosis (Table 2).

Univariate analysis (Table 3) showed treatment modality with an esophageal metallic stent (P = 0.007, figure 3A) and radiotherapy treatment after fistula diagnosis (P = 0.04, figure 3B) were predictive of superior overall survival. The median survival was 125 days in the 30 patients receiving an esophageal metallic stent and 55 days in the 35 patients receiving a feeding gastrostomy/jejunostomy. In multivariate comparison, treatment modality with an esophageal metallic stent (P = 0.026, odds ratio: 1.859, 95% confidence interval: 1.076–3.214) represented the independent predictive factors of superior overall survival in esophageal squamous cell carcinoma patients with TEF.

Of the 30 patients with an esophageal metallic stent, 25 patients received a single esophageal metallic stent, and 5 patients received double stenting (stents in both the esophagus and tracheobronchus) due to TEF being combined with upper airway stenosis. Of the 5 patients with double stenting, they all received airway prostheses first, then esophageal metallic stents. The median survival was 148 days in the 5 patients with double stenting, and 120 days in the 25 patients with single stenting. There was no significant difference (P = 0.40, data not shown).

Stent placement in the esophagus and airway was technically successful in all patients with no severe procedure-related complications, such as perforation, massive bleeding, respiratory failure, or mortality. 12 patients experienced post-procedural retrosternal pain and chest discomfort. One patient had foreign body sensations and another developed a mild upper gastrointestinal hemorrhage (bleeding rate: 3.3%). After stent placement, 25 (83%) and 26 (87%) patients experienced improvement of dysphagia and respiratory symptoms, respectively.

### Nutrition Status and Other Laboratory Data between Patients Treated with an Esophageal Metallic Stent and those with a Feeding Gastrostomy/Jejunostomy

Serum albumin data were available in 19 of the 30 patients who received an esophageal metallic stent and 22 of the 35 patients who received a feeding gastrostomy/jejunostomy. Compared to baseline serum albumin and body weight (within the week before fistula diagnosis), mean body weight in stenting and feeding gastrostomy/jejunostomy groups decreased 3–4 weeks after fistula diagnosis. There were no significant differences between stenting and feeding gastrostomy/jejunostomy groups in mean decrease in serum albumin (0.06 g/dL in the stenting group versus 0.17 g/dL in the gastrostomy/jejunostomy group, P = 0.21, figure 4A) or mean body weight loss (0.38 kg in the stenting group versus 1.1 kg in the gastrostomy/jejunostomy group, P = 0.66, figure 4B). Furthermore, other laboratory data including CBC and liver function were also compared between two groups. The CBC data were available in all patients, and the data of liver function test were available in 28 of the 30 patients who received an esophageal metallic stent and 26 of the 35 patients who received a feeding gastrostomy/jejunostomy. There were no significant differences between stenting and feeding gastrostomy/jejunostomy groups in white blood cell, hemoglobin, platelet, aspartate transaminase (AST), alanine transaminase (ALT), and total bilirubin (Figure 4C–4H).

### Chemotherapy within 30 Days after Fistula Diagnosis between Patients Treated with an Esophageal Metallic Stent and those with a Feeding Gastrostomy/Jejunostomy

To objectively compare the infection control rate between the two groups, we used “chemotherapy within 30 days after fistula diagnosis” as a surrogate marker for infection control because chemotherapy cannot be performed when an infection is not under control. Initial presentation with infection was found in 21 of the 30 patients (70%) in the stenting group, and 26 of the 35 patients (74%) in the feeding gastrostomy/jejunostomy group, respectively. There was no significant (P = 0.70) difference between groups in the rate of initial presentation with infection. There were 16 (53%) patients in the stenting group and 5 (14%) patients in the feeding gastrostomy/jejunostomy group receiving chemotherapy within 30 days after the diagnosis of fistula. Compared to the feeding gastrostomy/jejunostomy group, a significantly higher proportion of patients (53% versus 14%, P = 0.001) in the stenting group were able to receive chemotherapy within 30 days after fistula diagnosis, indicating better infection control in the stenting group.

## Discussion

A malignant tracheoesophageal/bronchoesophageal fistula (TEF) is a life-threatening complication of esophageal cancer that carries risk of lower respiratory tract infection, poor nutrition and high mortality. The large study by Martini et al. [2] in 1970 revealed that TEF occurred in 4.94% of 1943 esophageal cancer patients. In our study, the incidence of fistula formation was 8.51% (86/1011), which is higher than the incidence from Martini et al. A recent study by Balazs et al. [1] also showed that the incidence of fistula formation was about 10%. The fistulas in the majority of patients develop at the late stage of esophageal cancer, and the respiratory complications and critical condition of the patients make fistula diagnosis difficult. As significant improvements have recently been made in supportive care, the diagnosis rate of fistulas may increase. Therefore, fistula formation may occur more frequently than that in previously reports.

The treatment options and patient survival for malignant TEFs have been reported in several retrospective studies (Table 4). Nowadays, the common treatment modalities for patients with TEFs include feeding gastrostomy/jejunostomy, stenting, and bypass surgery. In comparison with feeding gastrostomy/jejunostomy, previous studies proved that successful stent prostheses implantation can improve quality of life [1], [5], [11]. However, to the best of our knowledge, the survival benefit of an esophageal metallic stent compared with the feeding gastrostomy/jejunostomy has not been well documented in the past. Hu et al. [5] showed that there is no significant difference in survival time between the stenting group and feeding gastrostomy group, but the number of patients is limited in this study. Our retrospective results revealed that an esophageal metallic stent can improve overall survival in patients with TEFs, in comparison with a feeding gastrostomy/jejunostomy. Our data may provide a rational base for using esophageal metallic stents for esophageal squamous cell carcinoma patients with TEF more frequently in the future.

Previous studies showed that low procedure-related complication incidence of 0–17% and mortality rate of 0–2% were the preferable reasons for esophageal metallic stent implantation [19], [20]. In our study, complications were extremely rare, and the mortality rate was 0%. Among the 30 patients who received an esophageal metallic stent, 11 patients were AJCC 7^th^ stage IV and 16 patients had received radiotherapy before stent implantation. Surgical palliation of TEFs, such as esophageal exclusion, esophageal bypass, or fistula resection and repair, may lead to high procedure-related risk, especially in patients with an advanced disease. Our retrospective results further support that implanting an esophageal metallic stent is a safe procedure despite patients having been heavily-treated and even at a late stage of the disease.

In the present study, 5 patients received double stenting due to TEFs combined with upper airway stenosis. The median survival of these 5 patients was 148 days, which was longer than that of the 25 patients who received a single esophageal metallic stent. However, there was no statistical significance, which may be attributed to the relatively small number of patients in the study. All 5 patients received airway prostheses first, then esophageal metallic stents without any severe procedure-related complications. It is recommended that the airway prosthesis should be placed first in order to avoid tracheal or bronchial compression secondary to the esophageal metallic stent. Application of double stenting has been proposed to yield better results than single stenting [21]. Further study is needed to confirm this finding.

The only disadvantage of an esophageal metallic stent in our country is its price. Esophageal metallic stents are not reimbursed by our health-insurance system, and esophageal squamous cell carcinoma is frequently associated with a lower socioeconomic level [22]. As compared with the best supportive care, our retrospective results showed that patients with feeding gastrostomy/jejunostomy had better prognoses. For those patients who cannot afford the cost of an esophageal metallic stent, feeding gastrostomy or jejunostomy may be an alternative choice.

In the present study, patients with TEF in the esophageal metallic stent group had better survival than those in the feeding gastrostomy/jejunostomy group. We tried to analyze the reasons for the better outcome in the stenting group, including nutrition status and infection control. With regard to nutrition status, we did not observe significant differences between the stenting and feeding gastrostomy/jejunostomy groups in either serum albumin change or body weight loss. Thus, the survival benefit may not be related to the better nutrition support in the stenting group. In other words, our retrospective results showed that an esophageal metallic stent can maintain patients’ nutrition status as well as feeding gastrostomy/jejunostomy. Siddiqui et al. [23] also reported that self-expanding silicone stents are an effective alternative to a feeding jejunostomy for maintaining nutrition during preoperative chemoradiotherapy in patients with esophageal cancer, which further supports our findings.

Furthermore, we have attempted to compare the infection control rate between the stenting and feeding gastrostomy/jejunostomy groups. Because our study was performed in a retrospective manner, we did not have detailed questionnaires for these patients. To objectively compare the infection control rate between the two groups, we used “chemotherapy within 30 days after fistula diagnosis” as a surrogate marker for infection control. It is well-known that chemotherapy cannot be performed if an infection is not under control. In the present study, the percentage of initial presentation with infection was similar between the two groups (70% versus 74%, P = 0.70). However, compared to the feeding gastrostomy/jejunostomy group, there were more patients in the stenting group who were able to receive chemotherapy within 30 days after fistula diagnosis (53% versus 14%, P = 0.001). This phenomenon may suggest that patients in the stenting group had better infection control than those in the feeding gastrostomy/jejunostomy group, which may contribute to the better survival rate in the stenting group. Previous studies [24], [25] also reported that self-expandable stents can protect the lung from continuous saliva and food contamination by occluding the fistula, and thus control the infection sooner in most patients.

Our study has important limitations. First, our results are based on the retrospective analysis from patients with TEF in Taiwan. Our database is limited to the variables that were collected for clinical management, and not all the patients had complete data. The patients’ symptoms and follow-up often depended on reviews of the medical records. Because esophageal metallic stents are not covered by Taiwan’s health-insurance system, patients in the stenting group may have higher socioeconomic status than those in the feeding gastrostomy/jejunostomy group. The retrospective design of this analysis further justifies the conclusion that a prospective study in the future is needed to define our findings. Second, we did not have detailed questionnaires for these patients and used “chemotherapy within 30 days after fistula diagnosis” as a surrogate marker for infection control. However, many different factors can be involved in making decision for chemotherapy. For example, poor wound healing after feeding gastrostomy/jejunostomy, bleeding after stent implantation, or patients own choice may postpone the chemotherapy.

In conclusion, an esophageal metallic stent could significantly improve the overall survival in patients with a malignant tracheoesophageal/bronchoesophageal fistula in our retrospective analysis. Therefore, we suggest that esophageal metallic stents may be considered as the first-line of treatment for patients with a malignant tracheoesophageal/bronchoesophageal fistula as long as there is no contraindication.
